# Does advance contact with research participants increase response to questionnaires: an updated systematic review and meta-analysis

**DOI:** 10.1186/s12874-021-01435-2

**Published:** 2021-11-27

**Authors:** Benjamin Woolf, Phil Edwards

**Affiliations:** 1 Department of Psychological Science, University of Bristol, 5 Priory Road, Bristol, UK; 2 Medical Research Council Integrative Epidemiology Unit, University of Bristol, Bristol, UK; 3 Faculty of Epidemiology and Population Health, London School of Hygiene and Tropical Medicine, London, UK

**Keywords:** Pre-notification, Systematic review, Questionnaire response

## Abstract

**Background:**

Questionnaires remain one of the most common forms of data collection in epidemiology, psychology and other human-sciences. However, results can be badly affected by non-response. One way to potentially reduce non-response is by sending potential study participants advance communication. The last systematic review to examine the effect of questionnaire pre-notification on response is 10 years old, and lacked a risk of bias assessment.

**Objectives:**

Update the section of the Cochrane systematic review, Edwards et al. (2009), on pre-notification to include 1) recently published studies, 2) an assessment of risk of bias, 3) Explore if heterogeneity is reduced by: delay between pre-contact and questionnaire delivery, the method of pre-contact, if pre-contact and questionnaire delivery differ, if the pre-contact includes a foot-in-the-door manipulation, and study’s the risk of bias.

**Methods:**

Inclusion criteria: population: any population, intervention: comparison of some type of pre-notification, comparison group: no pre-notification, outcome: response rates. Study design: randomised controlled trails. Exclusion criteria: NA. Data sources: Studies which cited or were included in Edwards et al. (2009); We additionally searched: CINAHL, Web of Science, PsycInfo, MEDLINE, EconLit, EMBASE, Cochrane Central, Cochrane CMR, ERIC, and Sociological Abstracts. The searches were implemented in June 2018 and May 2021. Study screening: a single reviewer screened studies, with a random 10% sample independently screened to ascertain accuracy. Data extraction: data was extracted by a single reviewer twice, with a week between each extraction. Risk of Bias: within studies bias was assessed using the Cochrane Risk of Bias tool (ROB1) by a single unblinded reviewer, across studies bias was assessed using funnel plots. Synthesis Method: study results were meta-analysed with a random effects model using the final response rate as the outcome. Evaluation of Uncertainty: Uncertainty was evaluated using the GRADE approach.

**Results:**

One hundred seven trials were included with 211,802 participants. Over-all pre-notification increased response, OR = 1.33 (95% CI: 1.20–1.47). However, there was a large amount of heterogeneity (I^2^ = 97.1%), which was not explained by the subgroup analyses. In addition, when studies at high or unclear risk of bias were excluded the effect was to reduced OR = 1.09 (95% CI: 0.99–1.20). Because of the large amount of heterogeneity, even after restricting to low risk of bias studies, there is still moderate uncertainty in these results.

**Conclusions:**

Using the GRADE evaluation, this review finds moderate evidence that pre-notification may not have an effect on response rates.

**Funding:**

Economic and Social Research Council.

**Preregistration:**

None.

**Supplementary Information:**

The online version contains supplementary material available at 10.1186/s12874-021-01435-2.

## Introduction

Questionnaires have been one of the most common methods of data collection across the social and medical sciences. For example, in epidemiology pen and paper questionnaires alone were used in 29.2% of over 2000 analytic epidemiological studies included in a review of articles published in high-impact medical journals between 2008 and 2009 [1]. Likewise, about a third of empirical research published in management and accounting journals use questionnaires, and a review of a top social psychology journal found that over 91% of empirical studies published in the second half of 2017 used some form of questionnaire [2, 3].

Inherent in using questionnaires is a risk of non-response. Potential participants, for example, might forget to complete questionnaires, and research ethics requires a right to refuse participation. Non-response can negatively impact on studies in three major ways: Firstly, non-response can introduce selection bias [4]. Secondly, even in the absence of selection bias, because non-response reduces the number of participants recruited into a study, non-response increases risk of random error (i.e. reduces statistical power and precision). Finally, non-response increases study costs [5].

It is therefore important to minimise non-response. One potential method is for the study team to contact potential participants in advance of them receiving the questionnaire (questionnaire pre-notification). In 2009, Edwards et al. published the third update of a 2003 Cochrane systematic review of randomised control trials evaluating methods of reducing non-response in both postal and electronic questionnaires [6]. They found that pre-contact increased response when compared to no pre-contact (OR = 1.5, 95% CI 1.26–1.78, for response after first questionnaire administration, and OR = 1.45, 95% CI 1.29–1.63 for response after final questionnaire administration). However, Edwards et al. (2009) did not assess the risk of bias in or across the included studies, and is now 10 years old, so therefore does not include research published in the last decade. In addition, there was substantial heterogeneity among the study results (*p* < 0.000001; I^2^ = 91% for the response after the first questionnaire administration, and *p* < 0.00001; I^2^ = 89% for the response after the final questionnaire administration).

There is therefore a need for an updated review which includes recently published studies, an assessment of bias risk in and across included studies. This review will:

1. Update Edwards et al. (2009)‘s systematic review and meta-analysis of randomised control trials examining the effect on non-response of pre-notification relative to no pre-notification (in any population) so that it includes papers published in the last decade.
2. To carry out an assessment of the risk of bias (i) in and (ii) across included studies.
3. To examine the extent to which between study heterogeneity is explained by: (A) the delay between pre-contact and questionnaire delivery, (B) method of pre-contact, (C) if pre-contact differs from questionnaire delivery, (D) if the pre-contact includes a foot-in-the-door manipulation (required participants to do something to receive the questionnaire), and (E) differences in the risk of bias of included studies, through conducting a subgroup analysis.

## Methods

### Protocol and registration

The methodology of the review and analysis was approved in advance by the LSHTM epidemiology MSc course directors. A copy of this form, approved on 21/03/2018, can be found in Supplementary Table 1. However the study was not otherwise registered.

This study received ethics approval from the London School of Hygiene and Tropical Medicine MSc Research Ethics Committee on 26/03/2018. This study has been written in accordance with PRISMA-2020 [7].

### Eligibility criteria

#### Inclusion criteria

- **Types of population**: This study followed Edwards et al. (2009) in using data from “[a]ny population (e.g. patients or healthcare providers and including any participants of non-health studies).” This should maximise generalisability over different contexts.
- **Types of interventions**: interventions must include some type of questionnaire pre-contact (pre-notification, advance letter/email/text/phone call or other co-referring term). No restriction is placed on the type of questionnaire pre-notification.
- **Comparison group**: Included studies need to be able to make a direct comparison of the effect of questionnaire pre-notification vs no pre-notification (i.e. include at least one arm which received identical treatment to the pre-notification arm other than not receiving the pre-notification).
- **Types of outcome measures**: The proportion or number of completed, or partially completed questionnaires returned after all follow-up contacts were complete.
- **Types of study design**: Any randomised control trial evaluating a method of advanced contact to increase response to questionnaires. The inclusion of only randomised control trials should on average eliminate risk of confounding biasing estimates within studies.

#### Exclusion criteria

There are no exclusion criteria.

### Information sources

Relevant studies identified by Edwards et al. (2009). A detailed description of the information sources, e.g. databases with dates of coverage, used in this study are in its methods section and Supplementary Tables, which can be freely accessed in the Cochrane Library (https://www.cochranelibrary.com/cdsr/doi/10.1002/14651858.MR000008.pub4/full).In addition, the references of all included studies, and any citation they, or Edwards et al. (2009), had received by the 28/6/2018 were checked for meeting the eligibility criteria.

The search strategy was developed by modifying the strategy used by Edwards et al. (2009), to make it more sensitive and specific to detecting studies examining questionnaire pre-notification, by adding terms denoting types of pre-notification, and removing terms relating to other methods. The strategy was validated by inputting the new terms into Google Scholar, and checking that it detected all relevant studies included in Edwards et al. (2009). The specific search terms are presenting in Supplementary Table 2. The search strategy was implemented in the same data-bases used in Edwards et al. (2009) from the date they were last searched till the present day. Specifically, the following databases were searched (with date restrictions in brackets): CINAHL (2007.12–2018.6); Dissertation & Thesis, Social Science Citation Index, Science Citation Index, and Index to Scientific & Technical Proceedings in Web of Science (2008.1–2018.6); PsycInfo (2008.1–2018.6); MEDLINE (2007.1–2018.6); EconLit (2008.1–2018.6); EMBASE (2008.1–2018.6); Cochrane Central (2008.1–2018.6); Cochrane CMR (2008.1–2018.6); ERIC (2008.1–2018.6); and Sociological Abstracts (2007.1–2018.6). After consultation with the LSHTM library, two databases searched by Edwards et al. (2009) (National Research Register and Social Psychological Educational Criminological Trials Register) were not searched because they were both deemed inaccessible and no longer operational**.** Any relevant reviews found in the literature search were examined for relevant studies.

.Finally, because the search was out of date, the search terms were re-implemented in CINAHL (2018.1–2021.5); Dissertation & Thesis, Social Science Citation Index, Science Citation Index, and Index to Scientific & Technical Proceedings in Web of Science (2018.1–2021.5); PsycInfo (2018.1–2021.5); MEDLINE (2018.1–2021.5); EMBASE (2018.1–2021.5). The search was not re-run in Cochrane Central, Cochrane CMR, ERIC, Sociological Abstracts, or EconLit because they accounted for only 2.5% of studies identified in a database in 2018.

Non-English papers were translated using Google Translate.

### Study selection

The eligibility assessment was conducted by one reviewer following a standardised procedure. This process was repeated on a random 10% by a second reviewer with 99.7% agreement. Citations were uploaded onto Covidence (http://www.covidence.org/), a website specially designed for paper screening by the Cochrane Collaboration. Covidence automatically identified duplicates of citation/abstracts, which were then manually checked for errors.

Studies were first screened based on abstracts and titles, then full text. This process was repeated for any study which was referenced by or itself cited by an included study, and on the content of any potentially relevant review identified in the search.

### Data collection process

A standardised data extraction sheet (Supplementary Table 3) was developed. The sheet was pilot tested on 10 randomly chosen studies from Edwards et al. (2009). One reviewer extracted data from included studies. To minimise transcription errors, this process was duplicated by the same reviewer 1 week later. Disagreements were resolved by extracting information for a third time and using the third extraction as the definitive extraction.

To check for duplication studies which shared at least one author were compared based on similarity of study population, date, and methodology. Duplicate trials were treated as a single study in the meta-analysis.

### Data items

Information extracted for each included trial comprised 5 domains:

1. Information on the inclusion criteria: The study design, nature of the control arm, information on the intervention arm(s), information about the outcome measurement (the number of responses, and/or the response rate, in each arm).
2. Information on risk of bias: how the allocation sequence was generated, information of allocation concealment, blinding of participants and personnel, blinding of outcome assessors, any incomplete outcome data, information on other possible sources of bias (e.g. source of funding).
3. Information on the participants: the total number of participants, numbers in each arm, setting, country.
4. Information on the outcome: number of items returned, or response rate, in each arm.
5. Other information: the time from the sending of pre-notification to questionnaire, if it includes a foot-in-the-door manipulation, the type of questionnaire administration, the type of pre-contact.

### Risk of bias in individual studies

Assessment of risk of bias within each study was conducted by one unblinded reviewer. Information on risk of bias was extracted twice with a one-week gap between each extraction, and conflicts were handled by using the results of a third extraction. Authors included in the 2018 search were contacted for extra information about study bias risk, and still existent copies of communication from Edwards et al. (2009) were examined.

Bias was evaluated using the Cochrane Risk of Bias tool [8]. The tool involves rating the risk of bias across 7 domains (random sequence generation, allocation concealment, blinding of participant and personnel, blinding of outcome assessment, incomplete outcome data, selective reporting, and other biases) at the outcome level. Within each domain, the studies were ranked as either high or low risk of bias, depending on the description of the study provided. If insufficient information was provided to form a decision, studies were designated as ‘unclear’ risk of bias. Studies were classified as at a low risk of bias if they had a low risk in all domains, at a high risk of bias if at a high risk in one domain, and were otherwise classified as having an unclear risk of bias. A full description of the tool can be found in chapter 8 of the Cochrane Handbook [8]. Results are stratified based on Risk of Bias score.

### Summary measures, and planned methods of results synthesis

The primary summary measure of association estimated was the ratio of the odds (OR) of response in the treatment groups compared with the odds of response in the control group.

In line with Edwards et al. (2009), the meta-analyses were performed by comparing the ORs using a random-effects model. The analysis was performed on an intention-to-treat basis. Outcomes were only included if they occurred within the period of follow up.

The results were synthesised in a meta-analysis conducted using STATA 15, using the ‘metan’ command [9]. To be consistent with Edwards et al. (2009), a random effects meta-analysis was used. Heterogeneity was assessed using the Cochran-Q Chi [2] statistical test for heterogeneity, and the I^2^ statistic [10]. Results were presented using a forest plot.

To test the hypothesis that heterogeneity is explained by 1) the length of time between pre-contact and questionnaire, 2) method of pre-contact, 3) if pre-contact and questionnaire delivery differ, 4) if the pre-contact includes a foot-in-the-door manipulation, four planned subgroup analyses were conducted by separately stratifying the meta-analysis on these factors. Studies in which participants were not all assigned to the same type of pre-notification were excluded.

### Risk of bias across studies

Risk of bias across studies was assessed with funnel plots. Asymmetry was investigated informally, by visually assessing how symmetrical the plots are around the effect estimate, and formally, using Harbord’s test. Funnel plots were created using the ‘metafunnel’ command in STATA. Because ORs are naturally correlated with their standard error, response rates were used instead of ORs [9].

### Assessment of certainty in the body of evidence

Outcome level limitations were evaluated using the GRADE approach [11] for both the overall estimate, and the estimate for studies at low risk of bias.

## Results

### Study section

**Fig. 1:**
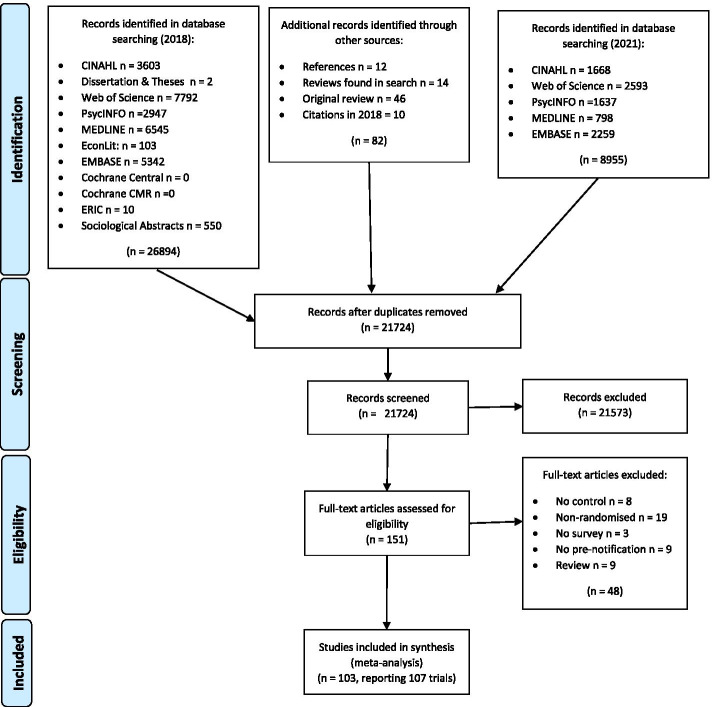
Flow diagram of study inclusion

A total of 103 papers, reporting a total of 107 trials, were identified for inclusion in the review. The search resulted in a total of 35,931 citations, including 14,207 duplications. Eight reviews (Supplementary Table 4) were included in the search and checked for citations. The reasons for exclusions are stated in Fig. 1 and Supplementary Table 5. The numbers identified and excluded at each stage are described Fig. 1. After re-reading the reports, and contacting study authors, five studies (Temple-Smith 1998 [12]; Waisanen 1954 [13]; Wright 1995 [14]; Wynn 1985 [15]) which were included in Edwards et al. (2009) were excluded for not having randomised participants to receive or not receive a pre-notification. No duplicates were identified during data extraction. Overall, the updated review now includes 60 more studies than Edwards et al. 2009; increasing the number of participants from 79,651 to 364,527.

### Study characteristics

Of the included studies, 32 (31.1%) were factorial designs. 60 (58.3%) were conducted in North America, 33 (32.0%) in Europe. Two (1.9%) were conducted in East Asia (Hong Kong and Thailand), 7 (6.7%) in Australia, one study did not state where it was conducted, and none were conducted in South America or Africa. 37 (35.9%) studies used samples of the general population. 13 (12.6%) were students or alumni, 14 (13.6%) were nested in other studies, 20 (19.4%) used medical or academic staff, 15 (14.5%) occupational samples, and 7 (6.7%) samples had some type of commercial basis. Approximately a third of questionnaires were health or epidemiology related. 6 (5.8%) trials were published prior to 1970, 8 (7.8%) in the 1970’s, 17 (16.5%) in the 1980’s, 20 (19.4%) in the 1990’s, 22 (21.4%) in the 2000’s, 28 (27.2%) in the 2010’s, and two (1.9%) in the 2020s. One study was not written in English.

85 (79.4%) of the pre-notifications were posted. 19 (17.8%) of the others were telephone, with a few delivered by email (*n* = 7, 6.5%) or text message (*n* = 7, 6.5%). Only 17 (15.9%) trials reported a pre-notification which included a foot-in-the-door manipulation. 28 (26.2%) trails had a delay of less than 1 week, 33 (30.8%) had a delay of 1 week, 11 (10.3%) of 2 weeks. One (0.9%) for delays of 3 weeks, 5 weeks and 6 weeks. 70 (65.4%) trails administered the questionnaire by mail, 24 (22.4%) over the phone, 12 (11.2%) by email or online, and one used interviews. The characteristics of the included studies are described in detail in Table 1.

**Table 1:** Full summary of included studies evaluating the effect of pre-notification on questionnaire response

Citation	Comparison	Outcome definition	Design	Setting (Country)	Topic	Delay Length	Pre-Contact Method	Survey Delivery	Foot-in-the-door?
Bergen 1957 [16]	Pre-contact or Control	final follow-up	experiment	primary school teachers (Netherlands)	options and attitudes towards public opinion researcher		mail	Postal	have to return a pre-paid return card
Albaum 1989 [17]	(Pre-contact or Control)x(leaflet or control)	final follow-up	factorial experiment	Business firms who do international market activities. (Denmark)	questions about work		mail	Postal	send description
Drummond 2008 [18]	(Pre-contact vs Control) x(questionnaire or control)	final follow-up	factorial experiment	GPs. (Ireland)	Health: views and practices about prostate-specific testing (PSA)	3 weeks	mail	Postal	none stated
Napoles-Springer 2004 [19]	Pre-contact or Control	final follow-up	Randomised control trail.	Nested in satisfaction survey of ambulatory care clinics. Have to use primary care and be older than 50 (USA)	about hospital experience year before/stratification	2 weeks	mail	Postal	none stated
Newby 2003 [20]	Pre-contact, Control, colour follow up, or monetary incentive	first and final follow-up	Randomised control trail.	random sample of business in Perth. exclude gov. enterprises and publicly owned firms (Australia)	about business: expectations and attitudes of the self employed	2 weeks	telephone	Postal	Asked relevant questions
Ogbourne 1986 [21]	Pre-contact or Control	first and final follow-up	Randomised control trail.	health and social workers (Canada)	about work		telephone	Postal (phone option in intervention)	offer a telephone interview if better than mail
Whiteman 2003 [22]	×2 types of incentives, Pre-contact or Control	first and final follow-up	Randomised control trail.	women age 40–60 and in Baltimore (USA)	women’s health	1 week	mail	Postal	none stated
Cycyota 2002 [23]	(Pre-contact or Control) x(incentive or cont.) x (personalisation or cont.) x(follow-up or cont.) x (postage or cont.)	final follow-up	factorial experiment	chamber of commerce survey to business (USA)	business climate	2 weeks	mail	Postal	none stated
Childers 1979 [24]	×2 types Pre-contact or Control	final follow-up	Randomised control trail.	agents of a large Midwest-based insurance company (USA)	insurance		mail	Postal	one group given return cards
Eaker 1998 [25]	(Pre-contact or Control) x (length or control) x (mention of telephone contact or control)	final follow-up	factorial experiment	Men and women living in Sweden in 1995 20–79 yrs. Old (Sweden)	health risk factors	1 week	mail	Postal	none stated
Etter 1998 [26]	(Pre-contact or control) x (layout or control)	first and final follow-up	factorial experiment	annual insurance questionnaire; residents of Geneva, valid address (Switzerland)	health insurance survey	2 weeks	mail	Postal	none stated
Ford 1967 A [27]	Pre-contact or control	first and final follow-up	experimental design	Residents of Chenoa (USA)	shopping survey	1 week	mail	Postal	none stated
Ford 1967 B [27]	Pre-contact or control	first and final follow-up	experimental design	Residents of Beardstown (USA)	shopping survey	1 week	mail	Postal	none stated
Hansen 1980 [28]	×2 Pre-contact, questionnaire length, or control	final follow-up	Randomised control trail.	People who bought cars in past year in Ohio (USA)	consumer’s attitudes towards recent new car purchases	3 days	telephone	Postal	Asked if willing to enter study.
Harrison 2004 [29]	Pre-contact or control	final follow-up	Randomised control trail.	patients referred to exercise referral scheme in past 12 months by primary care (	survey on relation between service expectations and outcomes	1 week	mail	Postal	none stated
Hornik 1982 [30]	Pre-contact or control	final follow-up	Randomised control trail.	Sample from telephone directly (USA)	about TV/advertising	under 1 week	telephone	Postal	none stated
Kephart 1958 [31]	Pre-contact or control	first and final follow-up	experiment	women who had taken state nursing exam in 1950 (USA)	Attitudes towards nursing profession	1 week	mail	Postal	none stated
Mann 2005 [32]	Pre-contact or control	final follow-up	experiment	registered voters in 3 states (USA)	election survey		mail	telephone	none stated
Parsons 1972 A [33]	Pre-contact or control	only one mailing	experiment	MBA alumni (USA)	politics and religion	4 days	mail	Postal	none stated
Parsons 1972 B [33]	Pre-contact or control	only one mailing	experiment	leaders of 2 religious sects (USA)	politics and religion	5 days	mail	Postal	none stated
Pirotta 1999 [34]	Pre-contact or control	first and final follow-up	Randomised control trail.	GPs. From health insurance In Victoria; have to have had 1500 consultations in prior year (Australia)	work	5 days	mail	Postal	ask for prompt return
Shiono 1991 [35]	Pre-contact or control	first and final follow-up	Randomised control trail.	physician who graduated from med school in 1985 (USA)	survey of pregnancy in physicians. Mailed was personalised	1 week	mail	Postal	toll free phone number to call if any questions about the survey
Spry 1989 [36]	Pre-contact or control × 3	first and final follow-up	factorial experiment	residence of San Diego (USA)	survey on health related behaviour. Enrolments from medical school director	under 1 week	phone or post card	Postal	none stated
Wiseman 1972 [37]	2 types of Pre-contact or control	final follow-up	experiment	residents of Boston (USA)	political issue polling	under 1 week	Telephone or mail	Postal	describes survey
Dillman 1974 [38]	Pre-contact or control	final follow-up	Randomised control trail.	sample of general public (USA)	feelings and concerns about Washington State University	na	telephone	Postal	ask questions to raise salience
Furst 1979 [39]	(Pre-contact or control) x (Personalisation or contorl)	final follow-up	factorial experiment	head teachers (USA)	personality test	under 1 week	mailed	Postal	none stated
Gillpatick 1994 [40]	(Pre-contact or control) x (2 types of monetary incentive or control)	only one mailing	factorial experiment	engineers who subscribe to a trade journal (USA)	market research (how good a CAD program is). One condition is personally pre-contacted, the other gets a referral from a colleague			Postal	none stated
Heaton 1965 [41]	Pre-contact or control	only one mailing	experiment	people who bought a Chevrolet in Philadelphia	car sales survey. Attempt to show importance of survey. Also personalised (e.g. hadn’t signed)	1 week	mailed	Postal	none stated
Jobber 1985 [42]	Pre-contact or control	final follow-up	Randomised control trail.	UK textile companies executives	Explore the design and extent of implementation of marketing information system		telephone	Postal	none stated
Jobber 1983 [43]	(Pre-contact or control) x (colour or control)	first and final follow-up	factorial experiment	UK textile companies	marketing practices		mailed	Postal	none mentioned
Kindra 1985 [44]	(Pre-contact or control) x (incentive or control)	first and final follow-up	factorial experiment	telephone directory (Canada)	response to advertising		telephone	Postal	Asked questions in pre-contact
Myers 1969 [45]	follow up, Pre-contact or control	only one mailing	Randomised control trail.	telephone directory (USA)	reaction to bank advertisement	1 week	mailed	Postal	none stated
Nichols 1988 [46]	Pre-contact or control)	final follow-up	Randomised control trail.	sample of electoral role (UK)	mailed was a leaflet on nutrition + cover mailed	5 weeks	mailed	Postal	none stated
Osborne 1996 [47]	Pre-contact or control	first and final follow-up	Randomised control trail.	GPs (Australia)	view on pathology test		telephone	Postal	none stated
Pucel 1971 [48]	Pre-contact or control	only one mailing	Randomised control trail.	graduates from 24 post-high schools (USA)	effect of training	1 week	mailed	Postal	none stated
Duhan 1990 [49]	Pre-contact or control	first and final follow-up	Randomised control trail.	marketing executives (USA)	work related	1 week	mailed	Postal	Asked questions
Faria 1990 [50]	×2 types of Pre-contact or control	first and final follow-up	Randomised control trail.	Homeowners residing on the property owners’ listing (USA)		under 1 week	phone or mailed	Postal	Asked if they will participate
Stafford 1966 [51]	×2 types of Pre-contact or control	only one mailing	Randomised control trail.	students (USA)	collegiate clothing	under 1 week	phone or mailed	Postal	none stated
Sutton 1992 [52]	(personalisation or control) x (Pre-contact or control)	first and final follow-up	factorial experiment	customers of a utility company, and contractors (USA)	reaction to an established energy rebate program	10–14 days: phone, 1 week: card	mailed	Postal	none stated
Taylor 1998 [53]	Pre-contact or control	first and final follow-up	Randomised control trail.	Young people in the Youth Cohort Study 8 sample, (UK)	Attitudes and behaviour	1 week	mailed	Postal	none stated
Martin 1989 [54]	(Pre-contact or control) x (follow up or control) x (personalisation or control) x (cover mailed or control) x (return postage or control)	final follow-up	factorial experiment	students in an urban university (USA)	views on the university		mailed	postal	none stated
Chebat 1991 [55]	(Pre-contact or control) x (incentive or control)	final follow-up	factorial experiment	The Quebec population within the legal driving age (Canada)				Postal	none stated
Xie 2013 [56]	Pre-contact or control	final follow-up	Randomised control trail.	female nurses, age 35–65. with correct contact information (Hong Kong)	work and health	1 week	mail	Postal	asked to send reply slip
Mitchell 2012 [57]	Pre-contact or control	final follow-up	Randomised control trail.	nested in follow up of the SCOOP clinical trial. Women aged between 70 and 84, at high risk of osteoporotic features (UK)	Trail questions	6 weeks	mail	Postal	none stated
Maclennan 2014 [58]	Pre-contact or control	first and final follow-up	Randomised control trail.	nested in RECORD clinical trial. Patients who had not responded to annual follow ups. Over 70, history of fracture, not in other methodological study (UK)	self-reported fracture and quality of life	≥ two weeks	telephone	Postal	none stated
Keding 2016 [59]	Pre-contact or control	only one mailing	Randomised control trail.	nested in ACUDep trial, primary care patients in N England. Have mobile phone	quality of life	4 days	sms	Postal	none stated
Hammink 2010 [60]	(Pre-contact or control) x (follow up or control)	first and final follow-up	factorial experiment	nested in survey of GP patients (Netherlands)	quality of care/experience	1 week	mail	Postal	none stated
Felix 2011 [61]	(Pre-contact or control) x (tone or control)	only one mailing	factorial experiment	authors of published maternal health research (UK)	applying their research to LIC	1 week	email	online	none stated
Bauman 2016 [62]	(Pre-contact or control) x (follow up or control)	first and final follow-up	factorial experiment	nested in 45 and Up Study. adults 45 to 100 living in New South Wales. (Australia)	socio-environmental causes of health	2 weeks	mail	Postal	none stated
Barra 2016 [63]	Pre-contact or control	first and final follow-up	Randomised control trail.	patients discharged in stated time who had not responded to previous survey, involved in other studies/care and had a phone number (Norway)	post stroke questionnaire	1 week	mail	Postal	ask for consent to receive survey
Bosnjak 2008 [64]	(×2 types Pre-contact or control) x (invitation or control)	final follow-up	factorial experiment	university students (Germany)	psychometrics, e.g. personality test	1 week	email or sms	online	none stated
Boyd 2015 [65]	(Pre-contact or control) ×2 follow up or control) x (design or control)	final follow-up	factorial experiment	ALSPAC follow up (UK)	consent to patients in follow up.	1 week	mail	Postal	none stated
Dykema 2011 [66]	(Pre-contact or control) x (incentive or control)	final follow-up	factorial experiment	physicians, (USA)	assess knowledge of genetic variation.	1 week	mail	online	none stated
Grande 2016 [67]	Pre-contact or control	only one mailing	Randomised control trail.	Random digit dialling (Australia)	epidemiological facts about the workplace	same day	sms	phone	none stated
Mclean 2014 [68]	(Pre-contact or control) x (design or control)	only one mailing	factorial experiment	the electoral roll (Australia)		1 week	mail	Postal	none stated
Rao 2010 [69]	(Pre-contact or control) x (follow up or control) x (incentive or control) x (recurrent mode or control)	final follow-up	factorial experiment	Random digit dialling	opinion poll survey	1 week	mail	Postal	none stated
Starr 2015 [70]	(Pre-contact or control) x (follow up or control)	final follow-up	factorial experiment	nested in epilepsy rct, provided phone number (UK)		under a week	sms	Postal	none stated
van Veen 2016 [71]	×2 types Pre-contact or control	only one mailing	Randomised control trail.	university students (Germany)	cheating in exams	1 week	mail	online	none stated
Ho-A-Yun 2007 [72]	Pre-contact or control	only one mailing	Randomised control trail.	GPs in Scotland (UK)		under a week	telephone	Postal	none stated but uses phone to call receptionist
Porter 2007 A [73]	(×2 types Pre-contact or control) x (follow up or control)	final follow-up	factorial experiment	high school students who contacted liberal arts college but did not apply (USA)	perceptions of college	1 week	email or post	online	none stated
Porter 2007 B [73]	(×2 types Pre-contact or control) x (follow up or control)	final follow-up	factorial experiment	alumni of liberal arts collage (USA)	career post-graduation	1 week	email or post	online	none stated
Atinc 2012 [74]	(Pre-contact or control) x (follow up or control)	final follow-up	factorial experiment	university staff and faculty (USA)		under a week	email	online	none stated
Walker 1977 [75]	Pre-contact or control	only one mailing	Randomised control trail.	credit card holders (USA)	consumer credit survey/purchase history	under a week	mailed	mail	none stated
Snow 1986 [76]	Pre-contact or control	final follow-up	Randomised control trail.	People existing job training partnership program (USA)	outcome of state-wide job training program	1.5 weeks	mailed	phone	none stated
Pitiyanuwat 1991 [77]	(Pre-contact or control) x (deadline or control) x (design or control)	first and final follow-up	factorial experiment	public school teachers, (Thailand)	desirable characteristics of a teacher		mailed	mail	none stated
Nicolaas 2015 [78]	(Pre-contact or control) x (follow up or control) x (design or control)	final follow-up	factorial experiment	embedded in a GP patient survey: over 18, registered with GP for >6mths (UK)	expense of patients of the NHS	1 week	mailed	mail	none stated
Link 2005 [79]	Pre-contact or control	only one mailing	Randomised control trail.	House holds in Behaviour Risk Factor Survey (USA)	health behaviours	under a week	mailed	phone	none stated
Kulka 1981 [80]	(Pre-contact or control) x (incentive or control) x (extra follow up or control) x (postage or control)	final follow-up	factorial experiment	registered nurses enrolled in a survey (USA)		2 weeks	mailed	mail	none stated
Kaplowitz 2004 [81]	Pre-contact or control	final follow-up	Randomised control trail.	university students (USA)			mailed	email	none stated
Groves 1987 [82]	X3 types of Pre-contact or control	only one mailing	Randomised control trail.	nested in NHIS survey (USA)	Health care		mailed	phone	none stated
Furse 1981 [83]	Pre-contact, incentive or control	final follow-up	Randomised control trail.	Tennessee population (USA)			phone	mail	none stated
Chebat 1993 [84]	(questionnaire type or control) x (Pre-contact, incentive or control)	first and final follow-up	factorial experiment	(Canada)		2 weeks	mailed	mail	none stated
Boser 1990 [85]	(Pre-contact or control) x (follow up or control)	final follow-up	factorial experiment	Graduates (USA)	emphasise value of participation	1 week	mailed	mail	none stated
Bergsten 1984 [86]	Pre-contact or control	only one mailing	Randomised control trail.	Medicare beneficiaries 65+ (USA)	access to health care	1 week	phone	interview	arrange time for interview
Baulne 2009 [87]	Pre-contact or control)	only one mailing	Randomised control trail.	people over 15 and live in a household. (Canada)	health		mail	phone	none stated
Henri 2012 [88]	X2 types Pre-contact or control	final follow-up	Randomised control trail.	listed companies (Canada)	management accounting research	2 weeks	telephone or mail	mail	none stated
Lalasz 2014 [89]	Pre-contact or control	final follow-up	Randomised control trail.	alumni 1 year post graduation (USA)	graduate careers etc.	2 weeks	mail	online	none stated
von der Lippe 2011 [90]	Pre-contact or control	only one mailing	Randomised control trail.	nested in German Health Update Survey 2009.	health	2 weeks	mail	phone	none stated
Lusinchi 2007 [91]	Pre-contact or control	first and final follow-up	Randomised control trail.	electrical engineers (USA)		under a week	email	online	none stated
McCallister 2008 [92]	X2 types Pre-contact or control	first and final follow-up	Randomised control trail.	university staff (USA)		under a week	post or email	mail	none stated
Miner 1983 [93]	X2 types Pre-contact or control	final follow-up	Randomised control trail.	parents/carers of people who had used a child psychiatry unit (USA)		under a week	post or phone	mail	none stated
Mitchell 2012 [57]	(Pre-contact or control) x (follow up or control)	only one mailing	factorial experiment	Academics in Northern UK		6 weeks	mail	mail	none stated
Steeh 2007 [94]	sms + called back (passive), sms + user has to call (active) or control	only one mailing	Randomised control trail.	Nexel subscribers. (USA)		Under 1 week	sms	phone	one treatment group have to call rather than be called to do interview
Vogl 2018 [5]	Pre-contact or control	only one mailing	Randomised control trail.	Random digit dialling (Germany)	Partner violence	1 week	mail	phone	none stated
Woodruff 2006 [95]	Pre-contact or control	final follow-up	Randomised control trail.	Random digit dialling (USA)	info about study/importance of patients. Parents/children for NIH study on teenage health	2 weeks	mail	phone	none stated
Traugott 1993 [96]	Pre-contact or control	only one mailing	Randomised control trail.	Random digit dialling (USA)			mailed	phone	none stated
Traugott 1987 [97, 98]	(Pre-contact or control) x (personalisation or control)	only one mailing	factorial experiment	Random digit dialling (USA)			mailed	phone	none stated
Brehm 1994 [99]	inactive + Pre-contact, Pre-contact, logo, or control	only one mailing	Randomised control trail.	Random digit dialling (USA)	Attitudes to war	1–2 weeks	mailed	phone	none stated
Camburn 1995 [100]	X3 types Pre-contact or control	final follow-up	Randomised control trail.	Random digit dialling (USA). Non house hold and non-working numbers excluded	child immunisation		mailed	phone	none stated
Dillman 1976 [101]	Pre-contact or control	only one mailing	Randomised control trail.	Random digit dialling (USA)			mailed	phone	none stated
Eyerman 2003 [102]	Pre-contact or control	only one mailing	Randomised control trail.	Random digit dialling (USA)	Health risk factors	under a week	mailed	phone	none stated
Goldstein 2002 [103]	Pre-contact or control	final follow-up	Randomised control trail.	Random digit dialling (USA)	Political polling	under a week	mailed	phone	none stated
Hembroff 2005 [104]	X2 types Pre-contact or control	final follow-up	Randomised control trail.	Random digit dialling (USA)			Mailed	phone	none stated
Iredell 2004 [105]	Pre-contact or control	final follow-up	Randomised control trail.	electoral roll. Have to be over 60 (Australia)	road crossing behaviour	2 weeks	mailed	phone	none stated
Mickey 1999 [106]	(Pre-contact or control) x (survey administration or control)	final follow-up	factorial experiment	over 40 (USA)		one week	mailed	in person or phone	none stated
Smith 1995 [107]	Pre-contact or control	only one mailing	Randomised control trail.	Random digit dialling (Australia). Exclude if non residential household, or non English speaking	health questions		mailed	phone	none stated
Singer 2000 [108]	Pre-contact or control	only one mailing	Randomised control trail.	Random digit dialling (USA)	consumer attitudes		mailed	phone	none stated
Gerritsen 2002 A [109]	Pre-contact or control	final follow-up	Randomised control trail.	Random digit dialling (Netherlands)	meat consumption, non-commercial	one week	mailed	phone	none stated
Gerritsen 2002 B [109]	Pre-contact or control	final follow-up	Randomised control trail.	people who had not answered their phone	meat consumption, non-commercial	one week	answer phone message	phone	none stated
Brick 1997 [110]	Pre-contact or control	final follow-up	Randomised control trail.	Random digit dialling (USA)			mailed	phone	Asked questions
Goulao 2020 [111]	Pre-contact or control	final follow-up	Randomised control trail.	Scotland	dentist patients	under a week	Postal	Postal	none stated
Rodgers 2018 [112]	Pre-contact or control	final follow-up	Randomised control trail.	UK	RCT outcomes	under a week	Postal	Postal	none stated
Sakshaug 2019 [113]	Pre-contact or control	final follow-up & before	Randomised control trail.	Germany	companies	one week	Postal	Email	Had to send an email
von Allmen 2019 [114]	Pre-contact or control	final follow-up	Randomised control trail.	Switzerland	aneurysm repairs	under a week	Postal	Phone	none stated
Griggs 2019 [115]	Pre-contact or control	final follow-up	Randomised control trail.	USA	Add Health wave V	under a week	Postal	Online	none stated
Vogl 2019 [116]	Pre-contact or control	final follow-up	Randomised control trail.	Germany	Violence survey	under a week	Postal	Phone	none stated
Gooden 2021 [117]	Pre-contact or control	final follow-up	Randomised control trail.	UK	mental health survey	under a week	Postal	Postal	none stated

### Risk of bias within studies

**Fig. 2:**
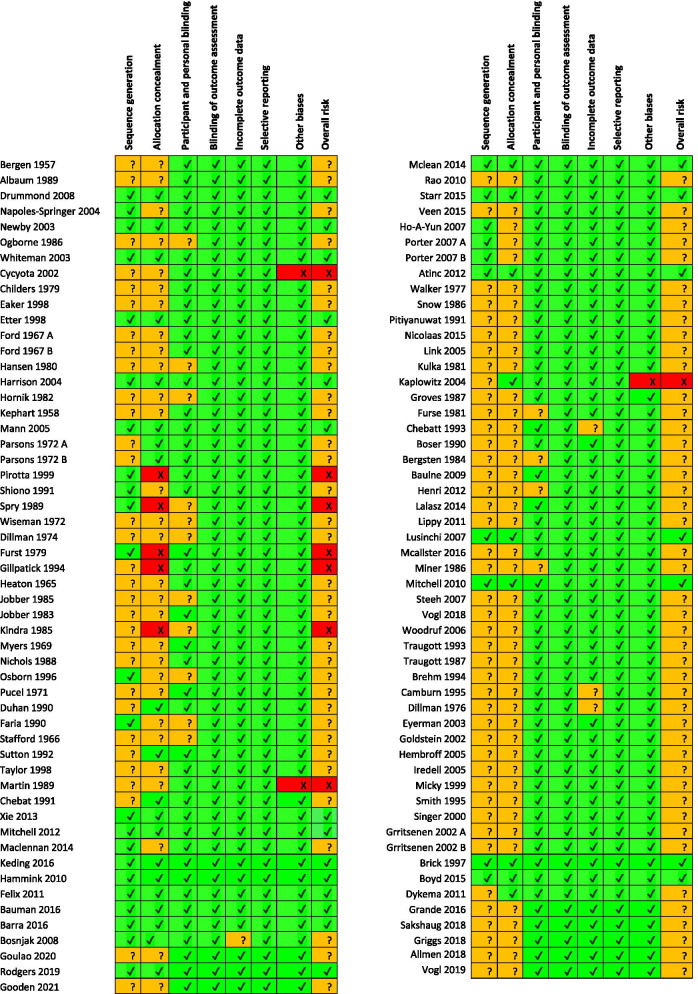
Risk of bias summary figure illustrating judgement about each risk of bias item for each included study

**Fig. 3:**
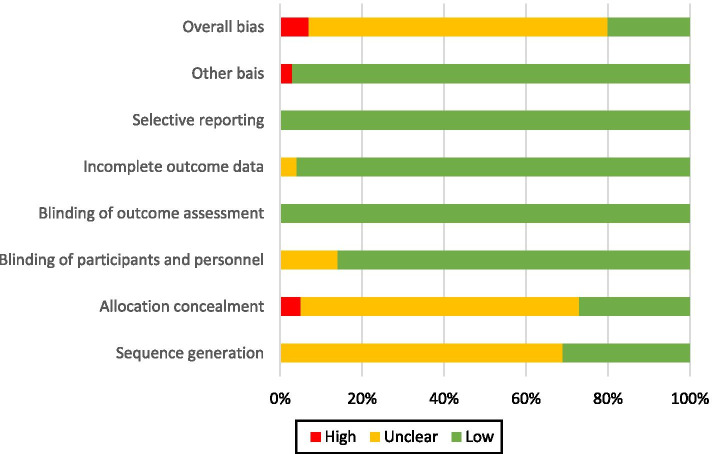
Risk of bias graph illustration judgments about each risk of bias item presented as percentages across all included studies

Judgments formed for each domain of the Cochrane Risk of Bias tool in each study are represented graphically in Fig. 2. The supporting evidence can be found in Supplementary Table 6. Overall, 8 studies were at high risk, 21 at low risk and 78 were at unclear risk. The proportions of studies at each level of risk is presented in Fig. 3.

#### Sequence generation

Thirty-three studies described the process used to generate the random sequence, or confirmed the use of randomisation in correspondence. Seventy-four studies have an uncertain risk of bias.

#### Allocation concealment

Thirty studies described concealment, or confirmed it in communication. Five confirmed that they had not used allocation concealment in communication. The remaining 72 studies provided insufficient information to reach a judgment, and so are of unclear bias.

#### Participant and personnel blinding

Participant and personnel blinding was not reported most trials. However, the design of many trials ensured that a degree of blinding did occur. A common design was to randomise participants to receive or not to receive a pre-notification without prior consent. The pre-notification itself would also often not explain that the participant had been allocated to receive it randomly. Thus any effect of treatment could not be due to the effect of knowing that they had been specially selected for an intervention which others had not got. Although the participant still knew they had received the pre-notification, this knowledge is part of the effect of a pre-notification – and therefore does not introduce any risk of material bias.

Similarly, although most did not describe any blinding procedure for personnel, its absence was often unlikely to lead to bias in estimates. In studies using a pre-written pre-contact (e.g. e-mail, letters, SMS) unblinded study personnel do not have the ability to influence the experience or perceptions of potential participants, as their only means of communication with each other is through a pre-written pro-forma message. This, however, is not true for studies which used a telephone pre-notification, in which the personnel and potential participants can have a genuine interaction. No study with telephone pre-notification reported no blinding of personnel.

Overall 92 studies were regarded as being at low risk of bias, and 15 at unclear risk.

#### Blinding of outcome assessment

Outcome assessment blinding was reported in 8 studies. However, the outcome (whether the questionnaire had been returned) is objective, and unlikely to be influenced by whether the outcome assessor knows the group assignment. Because the analyses are a comparison of two proportions, data analysers were unlikely to have enough researcher degrees of freedom for bias to be introduced in the analyses. All studies were therefore judged as being at low risk of bias for this domain.

#### Incomplete outcome data

One hundred three provided enough information to ascertain the total number of participants randomised in each arm and the total number of questionnaires returned in each arm. However, 4 are at unclear risk because they did not report sufficient detail to estimate per protocol rates, or state if the rates were intention to treat or per protocol, and one study at high risk.

#### Selective reporting

There was little evidence of selective reporting. All studies reported information on the relevant outcomes of interest. However, study protocols were not examined.

#### Other biases

Three of the factorial studies had significant interaction effects.

### Results of individual studies

**Fig. 4:**
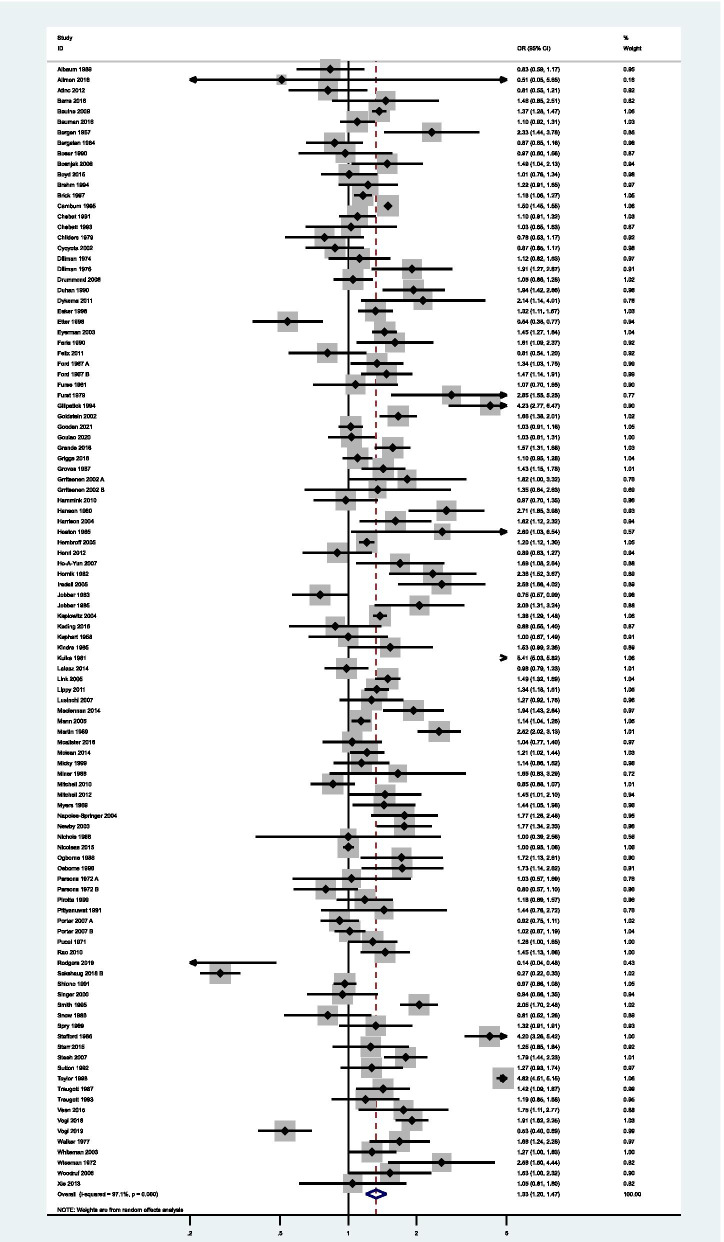
Forest plot of overall response after final follow-up with pre-notification versus no pre-notification

The results from individual studies are presented in a forest plot, Fig. 4. Fifty-nine studies had 95% confidence intervals which were incompatible with the null hypothesis, of which 55 implied that pre-notification increased response rates. There were a number of studies which appeared to have extreme results (Stafford 1966 [51]; Kulka 1981 [80]; Gillpatick 1994 [40]; Rodgers 2018 [112]; Sakshaug 2019 [113]; Taylor 1998 [53]). The extreme result of Rodgers appears to be due to the unusually high overall rate of response (97.1%). The other apparent outliers all were at high or unclear risk of bias.

### Synthesis of results

Information on response was available in all trials, thus data from all trials was used. These randomised a total of 338,429 participants, and had 174,323 returned questionnaires. The pooled estimate shows an increase in response for the final follow-up after questionnaire pre-notification (OR = 1.33, 95% CI: 1.20–1.47, *p* < 0.001), compared to an increase of 1.45 (95% CI 1.29 to 1.63) for Edwards 2009 (Supplementary Table 7). There was strong evidence of heterogeneity (I^2^ = 97.1%; Tau^2^ = 0.26; *Χ*^2^ (107, *N* = 107) = 3710.90, *p* < 0.001).

All subgroups, in the stratified meta-analyse, show significant amounts of heterogeneity (Supplementary Table 8). However, studies with low risks of bias and which send the pre-notification online had 95% confidence intervals which were compatible with the null hypothesis and appears to have reduced I^2^ (67.4 and 65.1% respectively).

### Risk of bias across studies

**Fig. 5:**
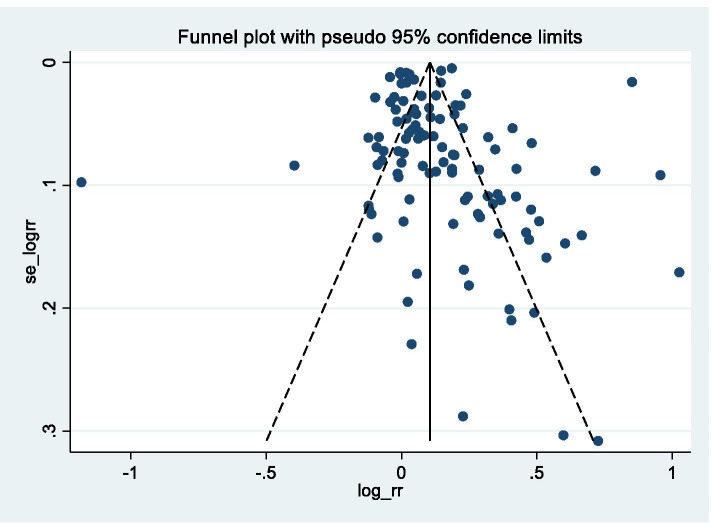
Funnel plot with pseudo 95% confidence limits for response after final follow-up

To explore the possibility of small study bias, funnel plots were created for the outcome, Fig. 5. Visual assessment implies that there is no major asymmetry. However, more studies than expected fell outside the 95% confidence limits. In addition, a formal assessment of asymmetry, using Harbord’s test, did not find evidence to reject the null hypothesis of no asymmetry (*p* = 0.749).

### Effect of risk of bias within studies on the pooled results

**Fig. 6:**
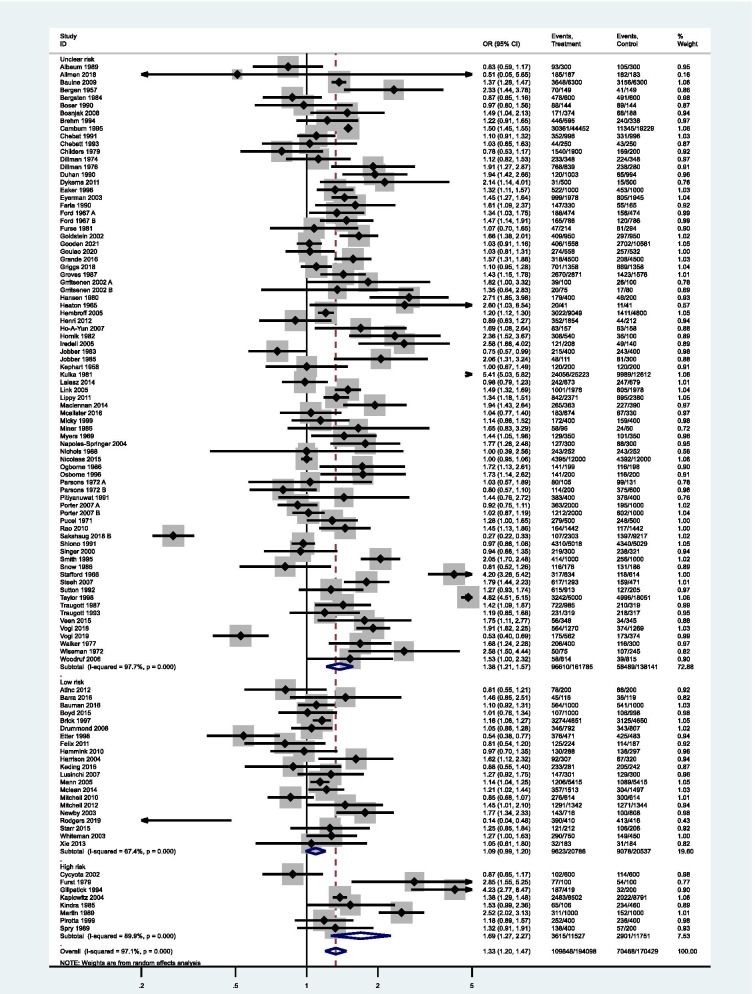
Forest plot of response after final follow-up with pre-notification versus no pre-notification, stratified by risk of bias

Seventy-eight studies were at unclear risk, 21 at low risk, and 8 at high. When stratified by risk of bias, there was no longer evidence against the assumption of a pooled association across studies which were of low bias (OR = 1.09, 95% CI: 0.99–1.20, Fig. 6).

### Assessment of certainty in the evidence

#### Risk of Bias

Across domains, high risk of bias was uncommon. However, few studies provided sufficient information to be assigned low risk of bias. The interpretation of the overall results is therefore downgraded.

#### Imprecision

Due to the large number of participants in each arm, even after stratification by bias risk, confidence intervals were relatively narrow. GRADE suggests additionally assessing he ‘optimum information size’ (i.e. have the number of participants a randomised trial needs to have sufficient power to answer the question) [118, 119]. Because larger sample sizes are required to detect smaller estimates, we calculated the optimum information size using information from the meta-analysis of studies at a low risk of bias (see Supplementary Table 7). Around 2500 participants would be required for each arm, for a 90% power and 5% alpha, which was obtained for both estimates.

#### Indirectness

There was generally little indirectness in the review. All studies were randomised control trials examining the effect of pre-notification on questionnaire response, so directly answered the review’s question.

#### Publication bias

Visual inspection of the funnel plots and formal testing with Harbord’s test both imply that small study bias was unlikely. As high questionnaire response is important to non-academics, e.g. polling companies, an unassessed grey literature will probably exist.

#### Heterogeneity

There was substantive heterogeneity within the review, and in all stratified analyses. We therefore downgraded the evidence due to the unexplained heterogeneity. Future studies should consider further explanations.

#### Overall GRADE evaluation

After two downgrades, there is low certainty in the overall estimate, but, with only one downgrade, moderate certainty in the estimate for studies at low risk of bias.

## Discussion and conclusions

### Summary and interpretation of evidence

This meta-analysis and systematic review of randomised control trials examined the effect of pre-notification compared to no pre-notification on questionnaire response rates. Pre-notification led to 1.33 (95% CI: 1.20–1.47) times greater odds for response. However, this was greatly reduced after restricting to studies of low risk of bias, OR = 1.09 (95% CI: 0.99–1.20).

This low OR implies that researchers should be cautious when using pre-notification as they may not lead to improvements in participant response rates. Specifically, in instances where pre-notification would be an expensive addition to a study, we believe that there is too much uncertainty to recommend the use of a pre-notification. One potential implication of the remaining unexplained heterogeneity is that there are unmeasured effect modifiers which cause pre-notification to work in some circumstances but not other. Therefore, if pre-notification would have a negligible impact on the cost of recruiting participants, nesting a high-quality randomised control trail could help reduce the uncertainty around the potential benefits of pre-notification in a specific setting.

### Limitations

#### Limitations of the evidence included in the review

##### Level of certainty in the evidence

The level of certainty in both the overall and low risk of bias estimates were downgraded because of high unexplained heterogeneity. Exploring other factors could be a topic of other reviews. The large number of high and unclear risk of bias studies lead to the overall estimate being downgraded an additional time.

The number of studies with an unclear risk of bias could have potentially been reduced if studies in the 2021 search were contacted for further information. However, the age of many of the remaining studies made communication difficult, e.g. due to address change, and information not being available for studies where contact could be made. In addition, between the beginning of the project and its end Cochrane released an updated version of the Risk of Bias tool. The new tool changed the structure of the evaluation and by allows reviewers to come to a qualitative decision about the probability of bias risk in each domain. Most studies with an unclear risk of bias have it because they did not describe randomisation and/or allocation concealment in sufficient detail. It is likely that many of these studies could have been either upgraded or downgraded when evaluated using ROB2 based of covariate balance. We would therefore expect fewer studies to have an unclear risk of bias if we had used ROB2.

##### Generalisability

There are very few studies from low- or middle-income countries. The review’s results may not generalise to any population, especially given the heterogeneous effect.

#### Limitations of the review process

##### Search strategy

Cochrane recommends that the literature searching be done by two independent reviewers, while this review only used one [120]. In addition, the search lacked specificity, and some extra publications might have been found by contacting authors to see if they had published other studies on the question. However, citation searching is not always common in systematic reviews, although it proved an effective way of detecting new studies.

##### Data extraction and risk of Bias assessment

Cochrane recommends that data extraction should be done by two independent reviewers [121]. Although this review only used one reviewer to extract data and conducted the risk of bias assessment, both were done twice by this reviewer, which should also reduce transcription errors. There is still, however, some risk of bias due to the reviewer being unblinded.

### Strengths and weaknesses in relation to other studies

The updated review more than doubled the number of included studies, even with four old studies were excluded for poor methodology (Supplementary Table 5). The overall results of the two studies are relatively similar, with overlapping confidence intervals overlap the results of the two studies might be consistent. However, restricting to low risk of bias studies implies that this estimate may be due to study bias. Therefore, while Edwards et al. (2009) concluded that pre-notification does improve response rates, this review would conclude that there is moderate evidence that pre-notification may not improve response rates to questionnaires.

Both Edwards et al., and this study, might be criticised for their choice of outcomes. Response rate does not entail response quality [5]. For example, a questionnaire might not have been fully completed, or completed inaccurately. In addition, to be a useful intervention for researchers pre-notification needs to be cost effective. However, neither of these outcomes are examined in the reviews.

The conclusion was also different from two other systematic reviews which explored a similar question. Both Lacy et al., and van Gelder et al., concluded that pre-notification did improve response rates (with OR = 1.45, 95CI 1.01 to 2.10, and OR = 1.12, 95%CI 1.12 to 1.22 respectively) [122–165]. However, the 95% CI of both of these studies is compatible with the results of this study, and neither of these studies stratified their metanalyses by risk of bias.

### Conclusions and implications for further studies and practice

This systematic review and meta-analyses of randomised control trials examining the effect of pre-notification on questionnaire response found evidence which supports the use of pre-notification. However, after excluding studies at high or unclear risk of bias the effect of the intervention was greatly reduced, and is probably no longer of relevance. The quality of evidence among low risk of bias studies was downgraded due to substantial unexplained heterogeneity. Future reviews could consider exploring other explanations. In addition, studies originated from a limited set of settings, such as generally high-income countries. Future studies could explore if the results generalise to new settings.

## Supplementary Information

**Additional file 1.**

**Additional file 2.**

**Additional file 3.**

**Additional file 4.**

**Additional file 5.**

**Additional file 6.**

**Additional file 7.**

**Additional file 8.**

## Data Availability

Data and materials not available in the paper will be made available though contacting the corresponding author.
