# Supplementary material for: Does advance contact with research participants increase response to questionnaires: an updated systematic review and meta-analysis
Source: BMC Med Res Methodol. 2021 Nov 27;21:265. doi: 10.1186/s12874-021-01435-2 (PMC8627623; doi:10.1186/s12874-021-01435-2)
Supplement: Supplementary file 2 — Additional file 2. [file 12874_2021_1435_MOESM2_ESM.docx]

Supplementary Table 2: Search Terms

| The following search terms were used to search in the databases stated above for potential studies, where ‘*’ represents truncation and ‘or’/’and’ represent the appropriate Boolean operators for the respective data base. No constraints were applied other than the stated time restrictions.   1. questionnaire 2. survey* 3. data collection 4. 1 or 2 or 3 5. respon* 6. return* 7. cooperat* 8. feedback 9. 5 or 6 or 7 or 8 10. control* 11. randomi* 12. blind* 13. mask* 14. trial* 15. compar* 16. experiment* 17. factorial 18. manipulation 19. 10 or 11 or 12 or 13 or 14 or 15 or 16 or 17 or 18 20. advance 21. advanced 22. earlier 23. prior 24. before* 25. already 26. afore 27. pre 28. previous 29. preceding 30. preliminary 31. introductory 32. primary 33. primer 34. foot-in-the-door 35. prompt* 36. prenotification 37. 20 or 21 or 22 or 23 or 24 or 25 or 26 or 27 or 28 or 29 or 30 or 31 or 32 or 33 or 34 or 35 or 36 38. letter 39. letters 40. postal 41. postcard 42. telephon* 43. telefon* 44. email* 45. mail* 46. leaflet* 47. phone 48. notice 49. remind* 50. communic* 51. contact 52. contacting 53. notification 54. foot-in-the-door 55. 38 or 39 or 40 or 41 or 42 or 43 or 44 or 45 or 46 or 47 or 48 or 49 or 50 or 51 or 52 or 53 or 54 56. 4 and 9 and 19 and 37 and 55 |
| --- |
