# Supplementary material for: Does advance contact with research participants increase response to questionnaires: an updated systematic review and meta-analysis
Source: BMC Med Res Methodol. 2021 Nov 27;21:265. doi: 10.1186/s12874-021-01435-2 (PMC8627623; doi:10.1186/s12874-021-01435-2)
Supplement: Supplementary file 3 — Additional file 3. [file 12874_2021_1435_MOESM3_ESM.docx]

Supplementary Table 3: Data Extraction Form

| ID |  |
| --- | --- |
| Intervention Arm(s) |  |
| Control Arm(s) |  |
| Outcome definition |  |
| Number in control arm(s) |  |
| Number in intervention arm(s) |  |
| Number/ rate of return in control arm |  |
| Number /rate of return in intervention arm |  |
| Total number of Participants |  |
| Length of data-collection period |  |
| Setting |  |
| Country |  |
| Questionnaire topic |  |
| Other design comments |  |
| Delay form pre-contact to questionnaire |  |
| Method of per-notification |  |
| Method of sending questionnaire |  |
| Foot-in-the-door? |  |
| Sequence generation |  |
| Allocation confinement |  |
| Participant and personnel blinding |  |
| Blinding of outcome assessment |  |
| Incomplete outcome data |  |
| Selective reporting |  |
| Other bias |  |
