# Supplementary material for: Does advance contact with research participants increase response to questionnaires: an updated systematic review and meta-analysis
Source: BMC Med Res Methodol. 2021 Nov 27;21:265. doi: 10.1186/s12874-021-01435-2 (PMC8627623; doi:10.1186/s12874-021-01435-2)
Supplement: Supplementary file 4 — Additional file 4. [file 12874_2021_1435_MOESM4_ESM.docx]

## Supplementary Table 4: Reviews found in the literature search and examined for relevant studies

| Edwards, P., Roberts, I., Clarke, M., DiGuiseppi, C., Pratap, S., Wentz, R., Kwan, I. and Cooper, R., 2007. Methods to increase response rates to postal questionnaires. *Cochrane database of systematic reviews*, (2).  Edwards, P.J., Roberts, I., Clarke, M.J., DiGuiseppi, C., Wentz, R., Kwan, I., Cooper, R., Felix, L.M. and Pratap, S., 2009. Methods to increase response to postal and electronic questionnaires. *The Cochrane Library*.  Lacey, R.J., Wilkie, R., Wynne-Jones, G., Jordan, J.L., Wersocki, E. and McBeth, J., 2017. Evidence for strategies that improve recruitment and retention of adults aged 65 years and over in randomised trials and observational studies: a systematic review. *Age and ageing*, *46*(6), pp.895-903.  Martins, Y., Lederman, R.I., Lowenstein, C.L., Joffe, S., Neville, B.A., Hastings, B.T. and Abel, G.A., 2012. Increasing response rates from physicians in oncology research: a structured literature review and data from a recent physician survey. *British Journal of Cancer*, *106*(6), p.1021.  Treweek, S., Mitchell, E., Pitkethly, M., Cook, J., Kjeldstrøm, M., Johansen, M., Taskila, T.K., Sullivan, F., Wilson, S., Jackson, C. and Jones, R., 2010. Strategies to improve recruitment to randomised controlled trials. *The Cochrane Library*.  Treweek, S., Pitkethly, M., Cook, J., Fraser, C., Mitchell, E., Sullivan, F., Jackson, C., Taskila, T.K. and Gardner, H., 2018. Strategies to improve recruitment to randomised trials. *The Cochrane Library*.  van Gelder, M.M., Vlenterie, R., IntHout, J., Engelen, L.J., Vrieling, A. and van de Belt, T.H., 2018. Most response-inducing strategies do not increase participation in observational studies: a systematic review and meta-analysis. *Journal of clinical epidemiology*, *99*, pp.1-13.  Weiner, M.D., Puniello, O.T. and Noland, R.B., 2016. Conducting Efficient Transit Surveys of Households Surrounding Transit-Oriented Developments. *Transportation Research Record: Journal of the Transportation Research Board*, (2594), pp.44-50.  Young, T. and Hopewell, S., 2011. Methods for obtaining unpublished data. *The Cochrane database of systematic reviews*, (11), pp.MR000027-MR000027. |
| --- |
