# Supplementary material for: Does advance contact with research participants increase response to questionnaires: an updated systematic review and meta-analysis
Source: BMC Med Res Methodol. 2021 Nov 27;21:265. doi: 10.1186/s12874-021-01435-2 (PMC8627623; doi:10.1186/s12874-021-01435-2)
Supplement: Supplementary file 7 — Additional file 7. [file 12874_2021_1435_MOESM7_ESM.docx]

| Review | Number of trials | n/N, treated | n/N, control | OR (95% CI) | I^2 | n/N, treated low risk of bias | n/N, control low risk of bias | OR low risk of bias (95% CI) | Conclusion |
| --- | --- | --- | --- | --- | --- | --- | --- | --- | --- |
| Edwards 2009 | 47 | 17870/34764 | 22897/44887 | 1.45 (1.29 – 1.63) | 89.02% | NA | NA | NA | Pre-notification increases response rates. |
| This review | 107 | 109848/194098 | 70468/170429 | 1.33 (1.20-1.47) | 97.1% | 9623/20786 | 9078/20537 | 1.09 (0.99-1.20 | Pre-notification may not increase response rates. |

Supplementary Table 7: comparison with Edwards 2009.
