# Supplementary material for: Does advance contact with research participants increase response to questionnaires: an updated systematic review and meta-analysis
Source: BMC Med Res Methodol. 2021 Nov 27;21:265. doi: 10.1186/s12874-021-01435-2 (PMC8627623; doi:10.1186/s12874-021-01435-2)
Supplement: Supplementary file 8 — Additional file 8. [file 12874_2021_1435_MOESM8_ESM.docx]

| Risk of Bias | | | | | | | | | | | | | | | | | | | | | | | | | | | | | | | | |
| --- | --- | --- | --- | --- | --- | --- | --- | --- | --- | --- | --- | --- | --- | --- | --- | --- | --- | --- | --- | --- | --- | --- | --- | --- | --- | --- | --- | --- | --- | --- | --- | --- |
| Low Risk of Bias | | | | | | | | | | | Unclear Risk of Bias | | | | | | | | | | | | High Risk of bias | | | | | | | | | |
| OR (95% CI) | | n/N, treatment | | | n/N, control | | I^2 (p-value) | | | | OR (95% CI) | | n/N, treatment | | | | n/N, control | | | I^2 (p-value) | | | OR (95% CI) | | n/N, treatment | | | n/N, control | | | I^2 (p-value) | |
| 1.09 (0.99-1.20) | | 9623/20786 | | | 9078/20537 | | 67.4% (<0.001) | | | | 1.38 (1.21-1.57) | | 96610/161785 | | | | 58589/138141 | | | 97.7% (<0.001) | | | 1.69 (1.27 – 2.27) | | 3615/11527 | | | 2901/11751 | | | 89.9% (<0.001) | |
| Delay between the sending of survey and pre-notification | | | | | | | | | | | | | | | | | | | | | | | | | | | | | | | | |
| Less than one week | | | | | | | | | | | One to two weeks | | | | | | | | | | | | Two or more weeks | | | | | | | | | |
| OR (95% CI) | | n/N, treatment | | | n/N, control | | I^2 (p-value) | | | | OR (95% CI) | | n/N, treatment | | | | n/N, control | | | I^2 (p-value) | | | OR (95% CI) | | n/N, treatment | | | n/N, control | | | I^2 (p-value) | |
| 1.53 (1.31-1.78) | | 5498/14911 | | | 4116/13914 | | 83.6% (<0.001) | | | | 1.29 (1.09-1.52) | | 56338/98510 | | | | 42104/107236 | | | 98.3%, <0.001 | | | 1.20 (0.76 – 1.87) | | 29776/37068 | | | 15096/22795 | | | 98.4 (<0.001) | |
| Method of sending pre-notification | | | | | | | | | | | | | | | | | | | | | | | | | | | | | | | | |
| Mail | | | | | | | | Email or online | | | | | | | | Telephone | | | | | | | | | SMS or text. | | | | | | | |
| OR (95% CI) | n/N, treatment | | n/N, control | | | I^2 (p-value) | | OR (95% CI) | | n/N, treatment | | n/N, control | | | I^2 (p-value) | OR (95% CI) | | | n/N, treatment | | n/N, control | | | I^2 (p-value) | OR (95% CI) | | n/N, treatment | | n/N, control | | | I^2 (p-value) |
| 1.27 (1.12-1.43) | 103890/ 177512 | | 66514/ 156772 | | | 97.7% (<0.001) | | 1.07 (0.86-1.33) | | 1293/3226 | | 808/1996 | | | 65.1% (0.009) | 1.73 (1.35-2.22) | | | 2641/5472 | | 2067/5002 | | | 84.9% (<0.001) | 1.60 (1.19 – 2.15) | | 1385/ 6473 | | 712/ 5563 | | | 78.4% (0.001) |
| Difference in the Methods of sending questionnaire and pre-notification. | | | | | | | | | | | | | | | | | | | | | | | | | | | | | | | | |
| Yes | | | | | | | | | | | | | | | | | | No | | | | | | | | | | | | | | |
| OR (95% CI) | | | | n/N, treatment | | | | | n/N, control | | | | | I^2 (p-value) | | | | OR (95% CI) | | | | n/N, treatment | | | | n/N, control | | | | I^2 (p-value) | | |
| 1.36 (1.25-1.48) | | | | 56469/109416 | | | | | 32572/84819 | | | | | 90.7% (<0.001) | | | | 1.23 (0.98-1.54) | | | | 49899/74905 | | | | 36283/80655 | | | | 98.4% (<0.001) | | |
| Foot in the door | | | | | | | | | | | | | | | | | | | | | | | | | | | | | | | | |
| Yes | | | | | | | | | | | | | | | | | | No | | | | | | | | | | | | | | |
| OR (95% CI) | | | | n/N, treatment | | | | | n/N, control | | | | | I^2 (p-value) | | | | OR (95% CI) | | | | n/N, treatment | | | | n/N, control | | | | I^2 (p-value) | | |
| 1.24 (0.92-1.68) | | | | 9224/15119 | | | | | 10236/22127 | | | | | 95.1% (<0.001) | | | | 1.34 (1.20 -1.50) | | | | 98467/17586 | | | | 59904/147631 | | | | 97.2% (<0.001) | | |

Supplementary Table 8: Results of Stratified meta-analyses
